# Effect of the HDAC Inhibitor on Histone Acetylation and Methyltransferases in A2780 Ovarian Cancer Cells

**DOI:** 10.3390/medicina57050456

**Published:** 2021-05-07

**Authors:** Umamaheswari Natarajan, Thiagarajan Venkatesan, Appu Rathinavelu

**Affiliations:** 1 Rumbaugh-Goodwin Institute for Cancer Research, Nova Southeastern University, Fort Lauderdale, FL 33314, USA; un15@nova.edu (U.N.); tvenkatesan@nova.edu (T.V.); 2 College of Pharmacy, Health Professions Division, Nova Southeastern University, Fort Lauderdale, FL 33314, USA

**Keywords:** SAHA, *p21^WAF1/CIP1^*, epigenetic alterations, histone modifications, DNA methyltransferase

## Abstract

*Background andObjective:* Epigenetic modifications are believed to play a significant role in the development of cancer progression, growth, differentiation, and cell death. One of the most popular histone deacetylases inhibitors (HDACIs), suberoylanilide hydroxamic acid (SAHA), also known as Vorinostat, can directly activate *p21^WAF1/CIP1^* gene transcription through hyperacetylation of histones by a p53 independent mechanism. In the present investigation, we evaluated the correlation between histone modifications and DNA methyltransferase enzyme levels following SAHA treatments in A2780 ovarian cancer cells. *Materials and Methods:* Acetylation of histones and methyltransferases levels were analyzed using RT^2^ profiler PCR array, immunoblotting, and immunofluorescence methods in 2D and 3D cell culture systems. *Results:* The inhibition of histone deacetylases (HDAC) activities by SAHA can reduce DNA methyl transferases / histone methyl transferases (DNMTs/HMTs) levels through induction of hyperacetylation of histones. Immunofluorescence analysis of cells growing in monolayers and spheroids revealed significant up-regulation of histone acetylation preceding the above-described changes. *Conclusions:* Our results depict an interesting interplay between histone hyperacetylation and a decrease in methyltransferase levels in ovarian cancer cells, which may have a positive impact on the overall outcomes of cancer treatment.

## 1. Introduction

Ovarian cancer (OC’s) is one of the most common gynecologic cancers and ranks as the sixth leading cause of death among women in the US. It is anticipated that approximately 21,410 new cases and 13,770 deaths will be reported in the US alone in 2021 [1]. The risk for developing ovarian cancer is high for women who are 40 years of age or more, and it increases with the addition of other risk factors such as breast cancer gene 1 or 2 (*BRCA1* or *BRCA2*) mutations, Lynch syndrome, endometriosis, obesity, late childbirth in life, and hormone therapy after menopause [2]. In general, the majority of OC patients are diagnosed in advanced stages due to a lack of specific symptoms; therefore, identification of reliable biomarkers would have great potential for detecting the disease early, which is vital for the successful treatment of OC. In the process of ovarian cancer development, epigenetic modifications are also known to contribute significantly. The two most commonly characterized epigenetic alterations are DNA methylation and histone modifications, which play an important role in the control of cell cycle arrest, cell differentiation, and cell death through regulation of gene transcriptions [3,4,5]. Specifically, histone acetyltransferases and methyltransferases (HATs/HMTs) are the important families of epigenetic enzymes that can covalently modify the histones involved in transcriptional regulation [6].

Histone acetylation occurs by the addition of an acetyl group (COCH3) to the lysine residues, which play an essential role in altering gene expression. While the addition of the acetyl group is catalyzed by the histone acetyltransferases/lysine acetyltransferases (HATs/KATs), the removal of acetyl groups from histones is catalyzed by histone deacetylase enzymes (HDACs) [7]. Consequently, HDAC inhibitors (HDACIs) have attracted a good deal of attention because they show broad-spectrum activity in altering epigenetically regulated cell growth mechanisms. The HDACIs from natural sources or synthetically derived chemical compounds can turn on genes through inhibition of HDAC activity and cause hyperacetylation [8]. HDACIs are reported to block cancer cell differentiation and induce cancer cell arrest and cell death through autophagy, apoptosis, necrosis, or necroptosis [8]. Therefore, HDACIs have generated significant enthusiasm in the field of oncology, with more than 40 clinical trials conducted so far. One of the popular HDACIs, suberoylanilide hydroxamic acid (SAHA also known as Vorinostat), was approved for the treatment of cutaneous T-cell lymphoma and is currently in clinical trials for the treatment of other types of cancers [9]. SAHA can directly activate *p21^WAF1/CIP1^* gene transcription, which is one of the most important cyclin-dependent kinase (CDK) inhibitors [10]. HDACI-induced hyperacetylation associated with the *p21^WAF1/CIP1^* promoter region is responsible for the transcriptional induction of the *p21^WAF1/CIP1^* gene and the consequent effects [11]. In ovarian cancer cells, SAHA has been reported to produce specific modifications in the pattern of acetylation and methylation of lysines in H3 and H4 histones that are associated with the *p21^WAF1/CIP1^* gene promoter [11]. On the other hand, the methylation of DNA by DNA methyltransferases (DNMTs) occurs in the fifth position of cytosine (5-methylcytosine or 5-mc), followed by guanines in the CpG islands of the mammalian DNA strands [12]. In normal cells, a very small amount of CpG di-nucleotides is methylated. Aberrant hypermethylation occurs at the CpG islands in the promoter regions during tumorigenesis [12]. DNMT1 encodes the maintenance methyltransferase, and DNMT3A/DNMT3B encodes the *de novo* methyltransferases, which are required to establish and maintain genomic methylation [13]. However, increased DNMT1 expression has been reported in several types of cancers, where a higher level of methylation was maintained by elevated enzyme levels [14]. In most cases, DNA methylation will have an opposite effect compared to acetylation and generally, it is expected to suppress gene expression. The methylation of histones mainly occurs on either lysine (K) or arginine (R) residues. Histone methylation is also required for various biological processes involved in the modulation of post-transcriptional regulation or gene silencing. For example, protein arginine N-methyltransferase 1 (PRMT1) is an important enzyme that plays a significant regulatory role in many biological processes, including carcinogenesis, and regulates the transcriptional activity of genes by modifying a series of substrates. The abnormal expression level of *PRMT1* has been seen with various cancer types such as lung, breast, prostate, colon, gastric, bladder, lymphoma, and glioma cancers [15], and it has been suggested that PRMT1 is critical for the development of ovarian carcinoma. Furthermore, another methyltransferase, suppressor of variegation 3–9 homolog 1 (SUV39H1), is the su(var)3–9, enhancer-of-zeste, trithorax (SET)-domain-containing histone lysine methyltransferase that catalyzes the trimethylation of *H3K9* (histone3 lys9), which is also known to play a significant role during cancer development. SUV39H1 performs an important role in the maintenance and establishment of heterochromatin structure through various mechanisms. Based on this property, SUV39H1 is assumed to be critical for heterochromatin stability and integrity.

A sufficient amount of evidence is now available in the literature to show that DNA methylation linked with histone modifications can profoundly affect the transcription of genes in cancer cells. Although it is not clear how the histone modifications may directly or indirectly alter DNA methylation, it can be anticipated that some other factors may be required to connect these two epigenetic alterations in cancer conditions [16]. Therefore, in the present investigation, we evaluated the correlation between histone modifications and DNA methyltransferase enzyme levels using HDACI (SAHA) treatments in A2780 ovarian cancer cells. Subsequently, the potential implications of this epigenetic cross-talk in controlling cancer cell growth were discussed. Altogether, our study suggested that the inhibition of HADC activities by SAHA can reduce DNMTs/HMTs levels through induction of hyperacetylation of histones. Though there are many reports available in the literature to show that DNMTs/HMTs are overexpressed in a wide variety of cancers, we provide strong evidence that the down-regulation of methyltransferases such as *DNMT3A*, *PRMT1*, *SUV39H1*, and *HDAC2* is linked to the hyperacetylation of H2A, H2B, H3, and H4 following SAHA treatment. Our results depict an interesting interplay between histone hyperacetylation and methyltransferase levels in ovarian cancer cells, which may have a positive impact on the overall outcomes of cancer treatment.

## 2. Materials and Methods

### 2.1. Cell Line and Reagents

The A2780 cell line was purchased from American Type Culture Collection (ATCC), and SAHA was purchased from Selleckchem (Houston, TX, USA). Primary antibodies against Ac-H2A, Ac-H2B, Ac-H3, Ac-H4, HDAC2, HDAC3, HDAC4, DNMT3A, PRMT1, SUV39H1, MDMX, p53, p21^WAF1/CIP1^, p27^Kip1^, AURKB, CDC25C, GADD45A (1:1000) and Alexa Fluor 488 dye were purchased from Cell Signaling Technology (Danvers, MA, USA). The MDM2 antibody (1:500) was purchased from Santa Cruz Biotechnology (Dallas, TX, USA). β-actin (1:5000), secondary antibodies (anti-rabbit, anti-mouse), horseradish peroxidase (HRP) conjugate, and dimethyl sulfoxide (DMSO) were purchased from Sigma Aldrich (St. Louis, MO, USA). Nitrocellulose membrane (NC) (0.45 µm) was purchased from Amersham (GE Healthcare Life Sciences, Marlborough, MA, USA) and KPL LumiGlo Reserve chemiluminescent substrate was purchased from SeraCare Life Sciences (Milford, MA, USA).

### 2.2. Cell Culture and Drug Treatments

A2780 cells were grown in Roswell Park Memorial Institute (RPMI) 1640 Medium. The growth medium was supplemented with 10% fetal bovine serum (FBS), 1% of amphotericin B, and penicillin-streptomycin. Cells were cultivated at 37 °C in a humidified atmosphere with 95% air, 5% CO_2_ and routinely passaged when they were 80–85% confluent. During experiments, the cells were treated with 7.5 µM SAHA for 24 h and used for cell viability determination by the trypan blue dye exclusion (TBDE) test. RNA and protein extraction was performed for PCR array and Western blot analysis, respectively.

### 2.3. Cell Viability Determination Using Trypan Blue Dye Exclusion (TBDE) Method

For the cell viability assessment, the A2780 cells were seeded at a density of 5000 cells/well in 96-well plates and incubated under 95% air and 5% CO_2_ at 37 °C in a humidified incubator for 24 h to allow them to adhere to the growth surface. Once the cells reached 80–85% confluency, they were treated with different concentrations of SAHA (0.5–10.0 µM) for 24 h. After incubation, cell viability was analyzed using the TBDE method. After removing the incubation medium, the cell suspension was mixed with equal amounts of 0.4% *w/v* TBD solution. The cells were allowed to incubate for 5 minutes at room temperature. The percentage of cell viability was quantified using the TC20 automated cell counter from Bio-Rad (Hercules, CA, USA).

### 2.4. RNA Extraction

Total RNA was extracted from A2780 cells by using an RNeasy mini kit according to the manufacturer’s protocol (Qiagen, Valencia, CA, USA). The total RNA concentrations and purity were determined by measuring the absorbance ratio at 260/280 nm. 500 ng of total RNA of control and SAHA-treated A2780 cells were transcribed into cDNA using an RT^2^ first strand kit. Both groups of cDNA were used for RT^2^ Profiler PCR Array analyses.

### 2.5. Human Epigenetic Chromatin Modification Enzymes RT^2^ Profiler PCR Array

The cDNA of control and SAHA (7.5 µM) treated A2780 cells were synthesized using the RT^2^ first strand kit in accordance with the company’s protocol, utilizing the respective total RNA extracts as the templates (Qiagen). Gene profiling was conducted using the Human Epigenetic Chromatin Modification Enzymes RT^2^ profiler PCR array (Catalog # PAHS-085Z, Qiagen). The relative level of gene expression was analyzed using the ∆∆CT method. The heat map generated from the RT^2^ profiler data shows a graphical illustration of fold changes by comparing the control and treatment groups. The heat map represents the least and most extreme gene expressions compared to the untreated A2780 cells.

### 2.6. Biological Pathway Analysis

In order to identify differentially expressed genes from the RT^2^ Profiler PCR array, data were further analyzed using the online software STRING (https://string-db.org/cgi/input.pl; accessed on 3 November 2020), which provides a research tool for collating gene interactions. The STRING database contains known and predicted protein-protein interactions, including direct (physical) and indirect (functional) associations and interactions aggregated from several primary databases.

### 2.7. Western Blot Analysis

After 24 h of SAHA treatments, the cells were lysed on ice for 30 min in radio-immunoprecipitation assay (RIPA) buffer with protease inhibitor cocktail and sodium orthovanadate (Santa Cruz Inc., Dallas, TX, USA) for protease and phosphatase inhibitions, respectively. According to the manufacturer’s instructions, the protein concentrations were assayed using the bicinchoninic acid (BCA) method (Thermo Fisher Scientific, Grand Island, NY, USA). For immunoblotting analysis, 25 μg of total cellular protein per lane were separated by 7.5, 10, 12, and 15% of SDS-PAGE and electrophoretically transferred onto NC membranes. After blocking with 5% skim milk powder in PBS with 0.3% Tween 20, the membranes were probed with specific primary antibodies for MDMX; MDM2; p53; p21^WAF1/CIP1^; p27^Kip1^; AURKB; CDC25C; GADD45A; cyclin D; acetyl form of H2A, H2B, H3, and H4; HDAC2; HDAC3; HDAC4; DNMT3A; PRMT1; SUV39H1; and β-actin. Finally, the detection of specific protein bands on the membranes was achieved using the enhanced chemiluminescent (ECL) reagent with a suitable substrate (Milford). The protein bands were imaged immediately using a UVP image analyzer (EC3 Chemi HR 410 Imaging System). The densitometric analyses were completed using the ImageJ (NIH, Bethesda, MD, USA) analysis tool.

### 2.8. Spheroid Formation

The A2780 cells (20,000 cells/mL) were seeded onto 24-well ultra-low attachment plates (Corning Inc., Lowell, MA, USA) in RPMI 1640 culture medium supplemented with 1% of B27 (Thermo Fisher Scientific) and incubated at 37 °C for 3 days for spheroid formation. The cells in the spheroids were pre-and post-treated with SAHA (7.5 µM) for 7 days. During the treatment period, the size and morphology of the spheroids were evaluated. As part of the assessment, the spheroids were also counted, and size measurements were made to determine spheroid volume using a Leica (DMI3000 B) microscope.

### 2.9. Immunofluorescence Analysis for 2D and 3D Culture

Monolayer cells and cells that formed the spheroids were permeabilized with ice-cold methanol (100%) at −20 °C for 15–30 min. The methanol-fixed 2D (monolayer) and 3D (spheroid) cell samples were blocked with 1% BSA in PBS with 0.5% Triton X-100 at 4 °C for 2 h. Following incubation, the monolayers and spheroids were washed with PBST and incubated with primary antibodies specific for p21^WAF1/CIP1^, Ac-H2A, Ac-H2B, Ac-H3, and Ac-H4 at 4 °C overnight. Following the incubation periods, the monolayer cells and spheroids were washed 2–3 times with PBST, then incubated with the Alexa-Fluor^®^ 488 conjugated secondary antibodies at room temperature for 1 h. Finally, immunofluorescence (green) images were acquired at 10× magnification and analyzed using a DMI3000 B Leica fluorescence microscope.

### 2.10. Statistical Analysis

All data are presented as the mean ± standard deviation (SD) values from at least three independent experiments. The statistical significance between the groups was examined using the one-way analysis of variance (one-way ANOVA) followed by Tukey’s honest significance test. A *p*-value representing less than 0.05 (*p* < 0.05) was considered statistically significant.

## 3. Results

### 3.1. The Effect of SAHA on Reduction of A2780 Cell Viability

**Figure 1:**
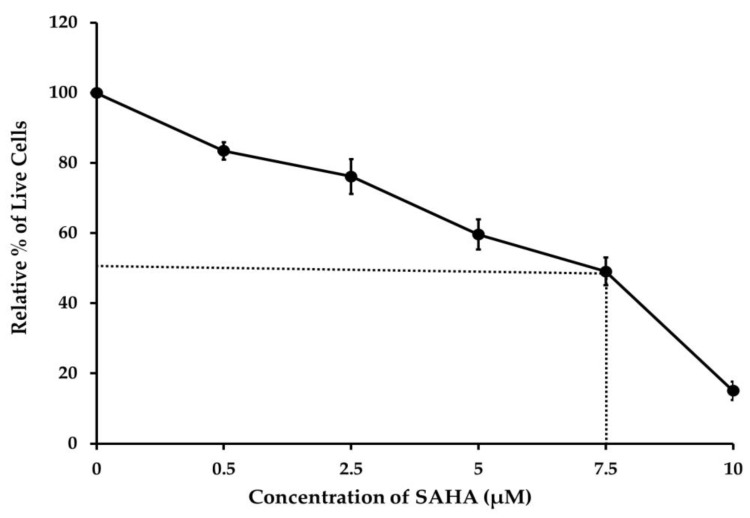
Ovarian cancer (A2780) cells were treated with different concentrations of suberoylanilide hydroxamic acid (SAHA) 0.5 to 10 µM for 24 h. Cell viability was analyzed by the trypan blue dye exclusion (TBDE) method. SAHA decreased the viability of A2780 cancer cells. Data are represented as mean ± SD from three independent experiments.

To determine whether the HDACI (SAHA) was inhibiting the growth of A2780 cells, we first assessed cell viability using the TBDE method. The results indicated that even though the cytotoxic effect started with a 0.5 μM concentration of SAHA, the treatment with 7.5 μM of SAHA was able to induce 50% cell death within 24 h (Figure 1). This experiment also demonstrated that 10 μM of SAHA caused nearly 80% of cell death compared to the control after 24 h of treatment. From this assay, the IC50 value of SAHA was calculated to be 7.5 μM for A2780 cells.

### 3.2. Identification of Differentially Expressed Genes in A2780 after SAHA Treatment Using Human Epigenetic Chromatin Modification Enzymes RT^2^ Profiler PCR Array

**Figure 2:**
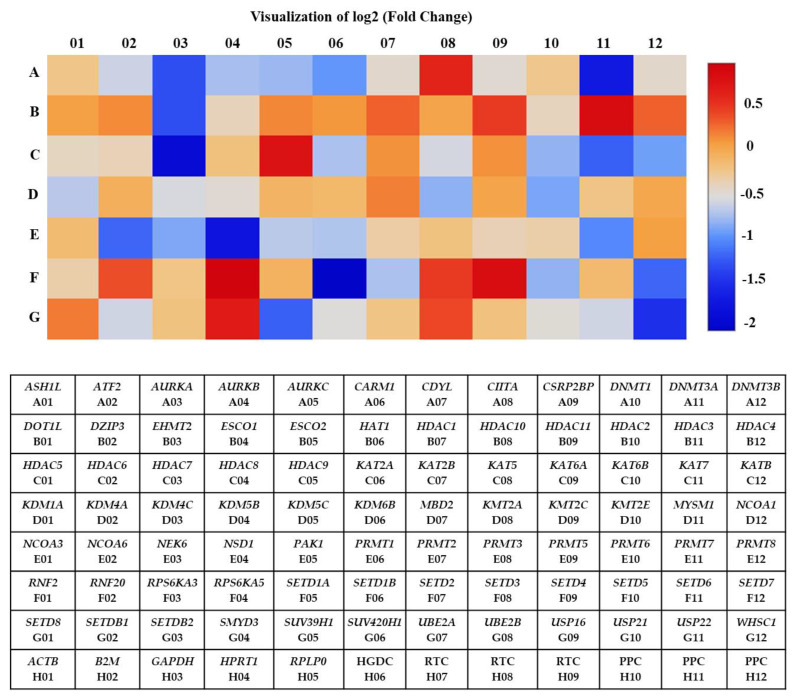
The heat map shows differentially expressed genes in A2780 cells after SAHA treatment. Red and blue color blocks represent high and low-level expressions, respectively. Human epigenetic chromatin modification enzymes gene table used in RT^2^ profiler PCR array experiments.

Total RNA extracted from A2780 cells treated with 7.5 µM of SAHA was analyzed compared to control using the RT^2^ profiler PCR array that was specific for the human epigenetic chromatin-modifying enzymes pathway. Out of the 84 genes analyzed by the PCR array, about 29 showed significant changes in their expression levels. Among the 29 genes, 23 showed down-regulation (˂1-fold), and 6 showed up-regulation (>1-fold) in their levels of expression compared to the untreated control (Table 1). A heat map was generated with a graphical representation of the fold changes between two groups and included the typical output: a blue signal representing minimum gene expression (down-regulation) and a red signal indicating maximum gene expression (up-regulation) compared to the untreated A2780 cells. The intensity of the indicated color is proportional to the degree of difference from the median. The heat map clearly shows that most of the genes were significantly down-regulated by SAHA treatment in A2780 cells (Figure 2). It is evident from our results that several genes involved in the chromatin-modifying enzymes pathway were significantly altered in their expression levels, compared to control cells, after treatment with SAHA for 24 h.

### 3.3. Network and Co-Expression Analysis

**Figure 3:**
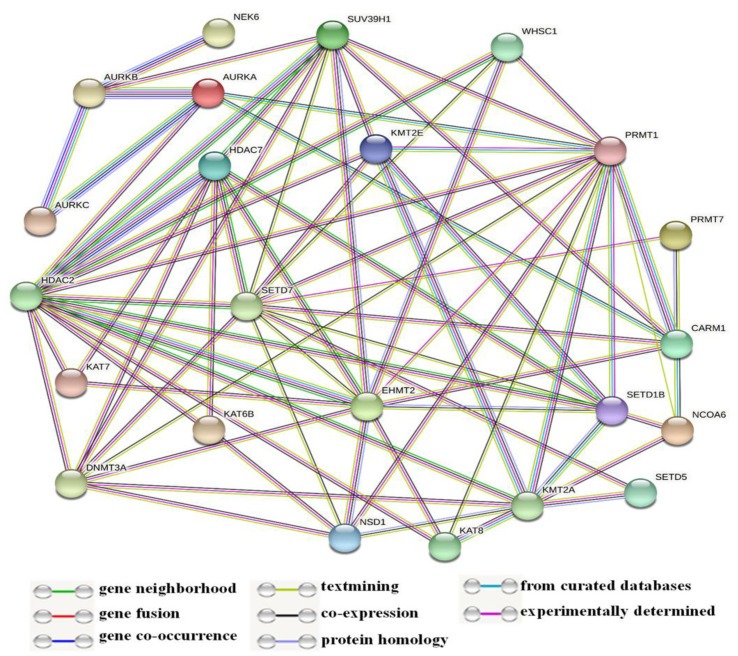
Illustration of network analysis of the protein associations. In this network, most of the genes are related to chromatin epigenetic modification enzymes.

**Figure 4:**
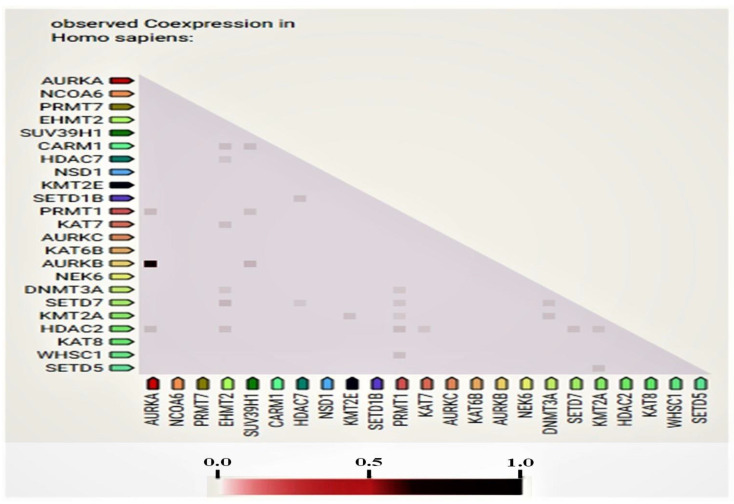
Proteins whose genes are observed to be correlated in expression across a large number of experiments. Co-expression scores based on RNA expression patterns and protein co-regulation provided by ProteomeHD.

In this study, some of the genes with a vital regulatory role in the epigenetics pathways and interactions with other genes were correlated using the online software STRING (Figure 3) for the network analysis. A total of 23 genes, including *SETD1B*, *HDAC7*, *NSD1*, *DNMT3A*, *WHSC1*, *AURKA*, *EHMT2*, *KAT7*, *SUV39H1*, *NCOA6*, *SETD7*, *PRMT7*, *CARM1*, *KAT8*, *KMT2E*, *NEK6*, *KMT2A*, *KAT6B*, *SETD5*, *AURKC*, *AURKB*, *PRMT1*, and *HDAC2*, were found to be prominently down-regulated. The network analysis revealed strong associations between *AURKB*, *AURKA*, and *AURKC* based on the experimentally determined data, gene co-occurrence, and gene co-expression status gathered from curated protein homology databases. On the other hand, *SUV39H1* showed associations with *AURKB*, *DNMT3A*, *PRMT1*, *CARM1*, *KMT2E*, *EMHT2*, *SETD7*, *HDAC2*, and *HDAC7.* Additionally, *PRMT1* and *HDAC2* displayed a strong association with many of the down-regulated genes in this study. In the co-expression analysis, *AURKB* exhibited a strong correlation with *AURKA*, with a score of 0.925. In addition, *DNMT3A* was found to be co-expressing with *EMH2* (0.045) and *PRMT1* (0.045); the scores were measured based on protein regulation. Furthermore, *SUV39H1* also showed an RNA co-expression score value with *AURKB* (0.150), *PRMT1* (0.074), and *CARM1* (0.085) in Homo sapiens. Thus, the network and co-expression analysis of the genes altered during SAHA treatment showed a strong network. Therefore, further exploring their relationships could lead to a better understanding of their mechanistic importance in the context of epigenetic alterations following HDAC inhibition (Figure 3 and Figure 4).

### 3.4. Up-Regulated Genes in the RT^2^ Profiler PCR Array Analysis

When we analyzed the gene expression profile in SAHA treated cells in comparison to the non-treated controls, we were able to see up-regulation in 6 genes, including *RPS6KA5*, *HDAC3*, *SETD4*, *HDAC9*, *SMYD3*, and *HDAC4*, which are directly involved in the epigenetic alterations. The increases of these gene expressions were 1.96, 1.79, 1.78, 1.70, 1.60, and 1.23 with a maximum increase (1.96 folds) observed for *RPS6KA5*, which is a kinase encoded by the *RPS6KA5* gene. This kinase, together with *RPS6KA4*, has been reported to mediate the histone H3 phosphorylation linked to the expression of immediate early genes. *SETD4* is a newly identified cytosolic and nuclear lysine methyltransferase involved in breast carcinogenesis. *SMYD3* encodes a histone methyltransferase that also plays a crucial role in transcriptional regulation as a member of an RNA polymerase complex.

### 3.5. The Effect of SAHA Treatments on the Regulation of A2780 Cell Cycle Arrest

**Figure 5:**
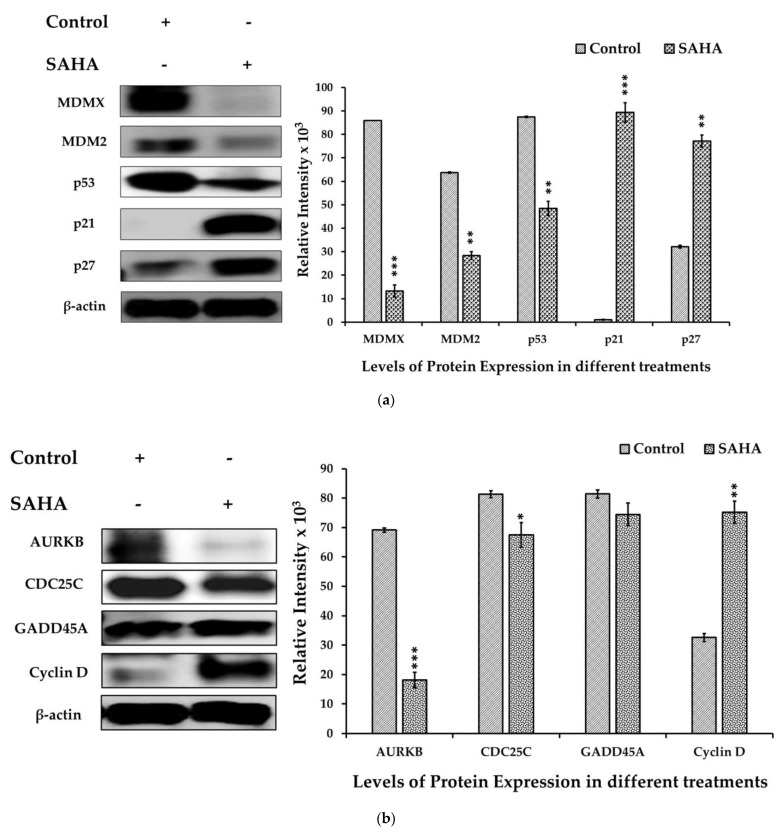
(**a**). SAHA induced cell cycle arrest in A2780 cells. The levels of cell cycle protein expression in the A2780 cells were measured by immunoblot analysis. MDMX, MDM2, and p53 expression levels were significantly decreased, and p21^WAF1/CIP1^ and p27^Kip1^ levels were increased in the SAHA (7.5 μM) treated cells compared with the untreated control. The bar graphs represent cell cycle-related protein levels following treatment with SAHA, as determined by densitometry of bands. All data are presented as the mean ± SD of at least 3 independent experiments. ** *p* < 0.01; *** *p* < 0.001. (**b**). Changes in the expression level of various cell cycle markers in A2780 cells. AURKB levels were significantly reduced, and CDC25C levels were also slightly diminished in SAHA (7.5 μM) treated cells compared with the control. The bar graphs represent other cell cycle-related protein levels following treatment with SAHA, as determined by densitometry of bands. All data are presented as the mean ± SD of at least 3 independent experiments. * *p* < 0.1; ** *p* < 0.01; *** *p* < 0.001.

To further confirm the intracellular effects of SAHA on A2780 cells, we analyzed the levels of several molecules involved in cell cycle progression after treatment with HDACI using Western blot analysis. As shown in Figure 5a, SAHA (7.5 μM) treated cells showed higher expression levels of p21^WAF1/CIP1^ and p27^Kip1^ than the control cells. In addition, SAHA-treated cells showed significant decreases in MDMX, MDM2, and p53 protein levels after 24 h of treatment. The results observed with the SAHA-treated A2780 cells were quite significant, because HDACI was able to increase both p21^WAF1/CIP1^ and p27^Kip1^ levels even though the MDMX, MDM2, and p53 levels were decreasing compared to the control A2780 cells. In addition, we observed a reduction in AURKB and CDC25C protein levels while cyclin-D levels were significantly elevated compared to the untreated control cells (Figure 5b). Thus, our results confirmed that treatment of A2780 cells with SAHA can elevate the levels of p21^WAF1/CIP1^ and p27^Kip1^ directly, and this elevation appears to be independent of p53.

### 3.6. The Effects of SAHA on the Acetylation of Histones and DNMTs/HMTs Levels in A2780 Cells

**Figure 6:**
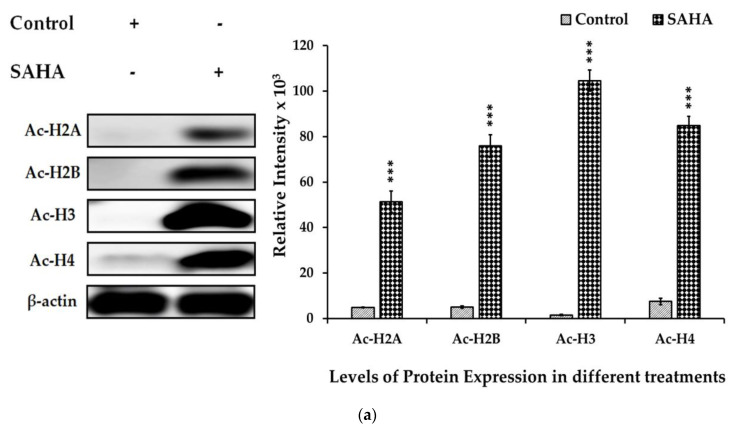

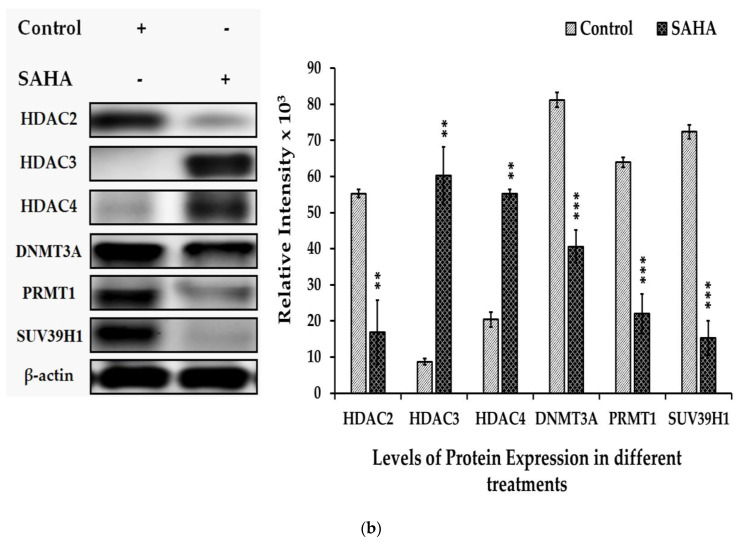
(**a**). Effects of SAHA on the expression of total acetyl form of histones. A2780 cells were treated with 7.5 µM of SAHA for 24 h. Western blotting was performed with the indicated level of acetylated histones such as H2A, H2B, H3, and H4. The bar graphs represent total acetylation of histones following treatment with SAHA, as determined by densitometry of bands. All data are presented as the mean ± SD of at least 3 independent experiments. *** *p* < 0.001. (**b**). Effects of SAHA on the expression of HDACs and DNMTs/HMTs in A2780 cells. Western blotting was performed to determine the levels of HDAC2, HDAC3, HDAC4, DNMT3A, PRMT1, and SUV39H1. The bar graphs represent HDACs and DNMTs/HMTs following treatment with SAHA, as determined by densitometry of bands. All data are presented as the mean ± SD of at least 3 independent experiments. ** *p* < 0.01; *** *p* < 0.001.

Epigenetic modifications such as acetylation and methylations can interact with each other to control cancer cell survival and proliferation. We examined the effect of SAHA (7.5 μM) on histone acetylation and DNMTs/HMTs levels in the A2780 cells. Our experiments showed that the acetylation of histones such as Ac-H2A, Ac-H2B, Ac-H3, and Ac-H4 was significantly increased (Figure 6a), while there was a notable decrease in methyltransferase levels following treatment with SAHA. Due to the suspected cross-talk between acetylation of histones and methylation, we examined the levels of HDAC2, HDAC3, HDAC4, DNMT3A, PRMT1, and SUV39H1 after SAHA treatment. Western blot analysis showed that SAHA significantly elevated the expression levels of HDAC3 and HDAC4. On the other hand, DNMTs/HMTs and HDAC2 expression levels were decreased significantly following HDACI treatment in A2780 cells (Figure 6b). Thus, our data confirmed that 7.5 μM of SAHA not only increased the acetylation of histone but also significantly reduced DNMTs/HMTs levels in A2780 cells.

### 3.7. Immunofluorescence Analysis of Acetylated Histones in A2780 Cells Growing as Monolayers and Spheroids

**Figure 7:**
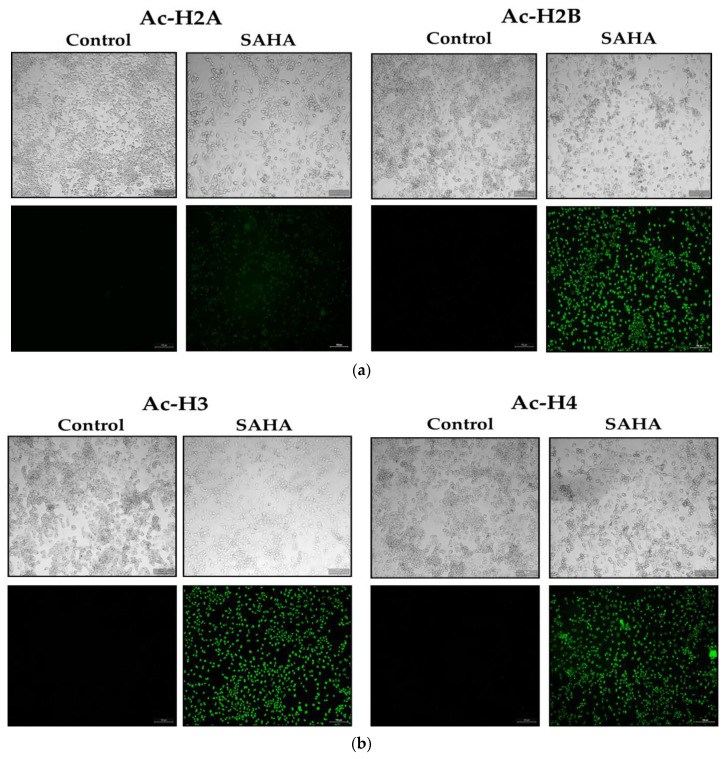

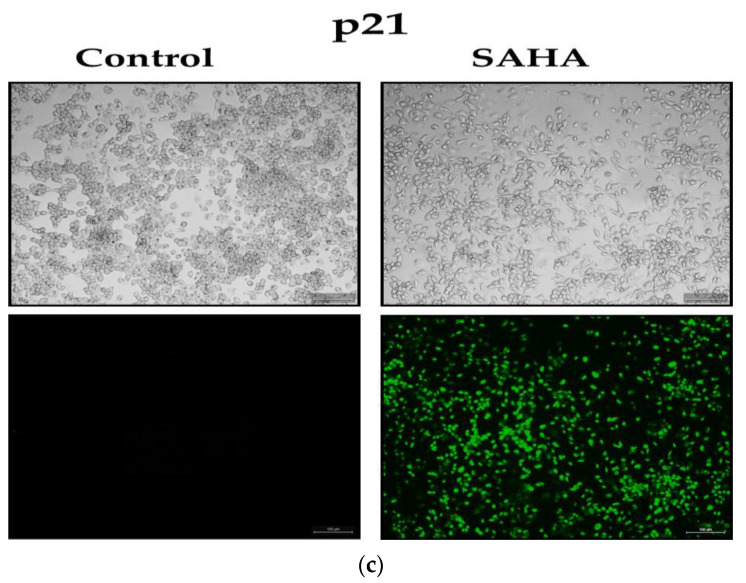
(**a**). A2780 monolayer cells stained for acetylated histones H2A and H2B. The light microscopic images of A2780 cells after SAHA (7.5 µM) treatment in the upper panels. The results of immunocytochemical detection of Ac-H2A and Ac-H2B are shown in the lower panels (green fluorescence) and visualized using Alexa Fluor^®^ 488. The monolayer cells were observed under Leica (DMI3000 B) microscope using 10× magnification. (**b**). A2780 monolayer cells stained for acetylated histones, including H3 and H4. The light microscopic images of A2780 cells after SAHA (7.5 µM) treatment in the upper panels. The results of immunocytochemical detection of Ac-H3 and Ac-H4 are shown in the lower panels (green fluorescence) and visualized using Alexa Fluor^®^ 488. (**c**). The A2780 monolayer cells of were stained for p21. The light microscopic images of A2780 cells after SAHA (7.5 µM) treatment in the upper panels. The results of immunocytochemical detection of p21^WAF1/CIP1^ levels are shown in the lower panels (green fluorescence) and visualized using Alexa Fluor^®^ 488.

To address whether the changes in the acetylated histones were associated with notable epigenetic alterations after SAHA treatment, we observed the effects of SAHA on cells growing in monolayers using the immunofluorescence detection method. We compared the expression levels of Ac-H2A, Ac-H2B, Ac-H3, Ac-H4, and p21^WAF1/CIP1^ following SAHA treatment in the monolayers of A2780 cells using immunofluorescence measurements (Figure 7a–c). After 24 h of treatment, SAHA was able to reduce the cell viability of the A2780 cells, which was evident from both the light microscopic and immunofluorescence imaging of the cancer cells. The increase in acetyl-histone levels correlated well with the increase in p21^WAF1/CIP1^ levels, as shown in Figure 7c.

### 3.8. Effect of SAHA Treatment on Spheroid Formation and Immunofluorescence Analysis of Acetylated Histones in A2780 Cells

**Figure 8:**
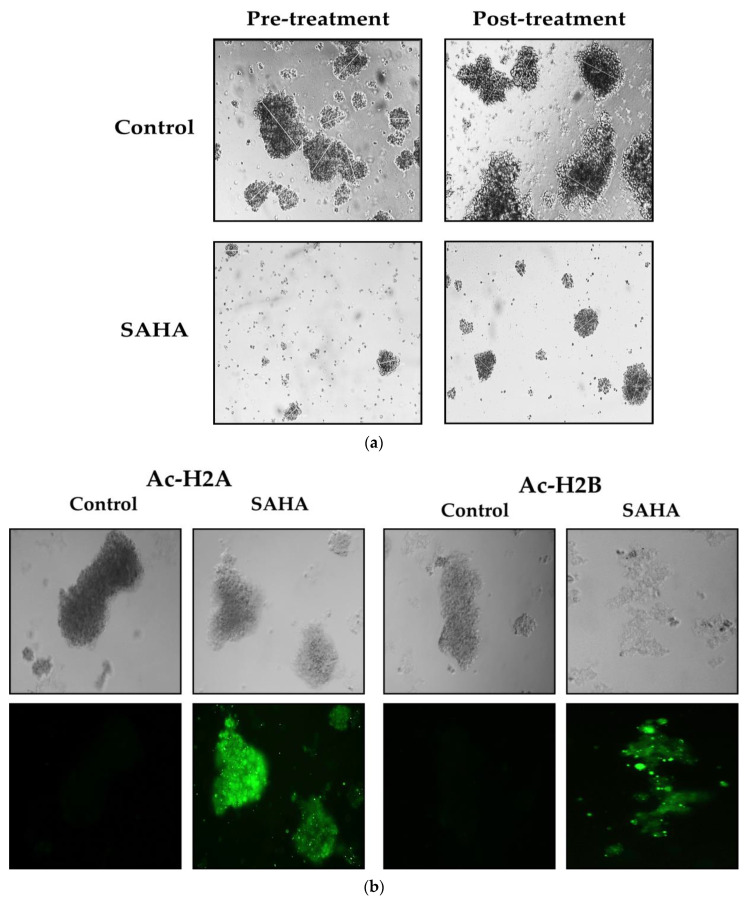

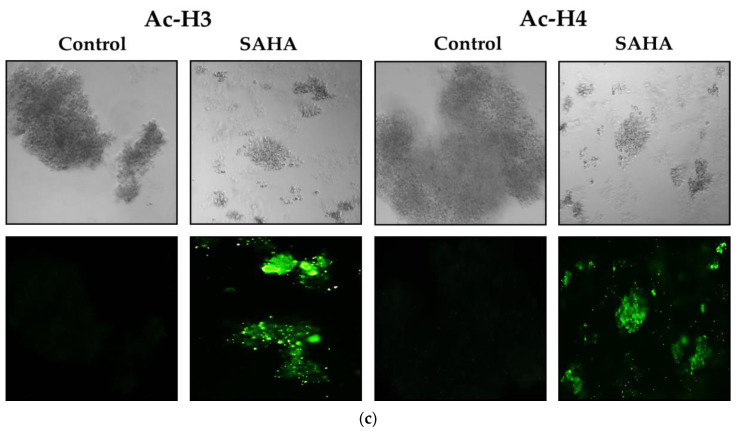
(**a**). HDAC inhibitor SAHA decreased the spheroid formation ability of A2780 cells in vitro. The morphology of A2780 cells treated with SAHA in the pre-and post- spheroid formation process, as observed using the Leica (DMI3000 B) microscope. (**b**). Spheroid formation of A2780 cells stained for acetylated histones. The spheroids were post-treated with SAHA for 7 days and then incubated with specific antibodies for Ac-H2A and Ac-H2B. Light microscopic images of A2780 cells after SAHA (7.5 µM) treatment in the upper panels. Immunocytochemical detection of endogenous period levels for Ac-H2A and Ac-H2B (green fluorescence), visualized using Alexa Fluor^®^ 488. (**c**). Spheroid formation of A2780 cells stained for acetylated histones. The spheroids were post-treated with SAHA for 7 days and then incubated with specific acetylated histones antibodies such as Ac-H3 and Ac-H4. Light microscopic images of A2780 cells after SAHA (7.5 µM) treatment in the upper panels. Immunocytochemical detection of endogenous spheroid levels for Ac-H3 and Ac-H4 (green fluorescence), visualized using Alexa Fluor^®^ 488. The spheroids were observed on a Leica (DMI3000 B) microscope at a magnification of 10×.

When A2780 cells were grown on ultra-low attachment plates in a serum-free culture medium that was supplemented with B27, they were able to colonize and form spheroids within 24 h after seeding. The cancer cells growing as spheroids survived longer than the cells growing in monolayers. During the culture periods, some cells died from serum deprivation, while others adapted to the low-serum environment and formed tiny 3D masses. When the A2780 cells formed dense spheroids, it was challenging to dissipate them by simple pipetting. Additionally, when the A2780 cells were grown in the B27 medium, they formed adenomatous cell clusters that spontaneously aggregated to form spheroids. Interestingly, it appears that HDACI inhibited spheroid size through two parallel mechanisms. During the growth phase, the spheroid diameters were monitored every day from day 0 to day 7 using a Leica microscope (DMI3000 B). In our experiments with 20,000 cells per well, spheroid diameters increased from 54 ± 16 μm to 349 ± 55 μm within 7 days in the control wells. The diameters and shapes of the spheroids formed by the untreated A2780 cells remained constant even after 14 days in comparison to the SAHA-treated cells, which formed spheroids with an average volume of 184 ± 8 μm only. Spheroid diameters decreased significantly (47 ± 8 μm) with SAHA treatment compared to the controls, to an average of 104 ± 21 μm (Figure 8a). These results showed that cells in 3D cell culture were also sensitive to SAHA treatment, similarly to the A2780 cells grown in a monolayer.

In regard to the acetylated histone levels in cells growing as spheroids, we were able to obtain results similar to the A2780 cells that were growing as monolayers. As shown in Figure 8b,c, after SAHA treatment, we were able to observe high levels of acetyl H2A, H2B, H3, and H4 in almost all the cells that had formed spheroids.

## 4. Discussion

Gene transcription activation and repression are controlled by a wide range of histone modifications that are enacted by histone modifiers and chromatin-bound proteins. Therefore, the right balance between specific changes and modifiers maintained at steady-state in the growing cell is necessary to maintain normal chromatin structure and execute proper gene expression programs. Once that balance is disrupted, cell phenotypes may be altered, and the cells may be primed for the onset of aberrant cell growth and progression [8]. Therefore, understanding the functions of the key regulators of histone modifications and uncovering the underlying alterations that impact the intracellular mechanisms will help us to develop drugs to maintain homeostasis and re-balance the mechanisms that disturb the cells. In this study, we primarily focused on analyzing the status of enzymes and proteins that are linked to acetylation and methylation of DNA and histones, which are known to be tightly associated with cancer cell growth.

**Figure 9:**
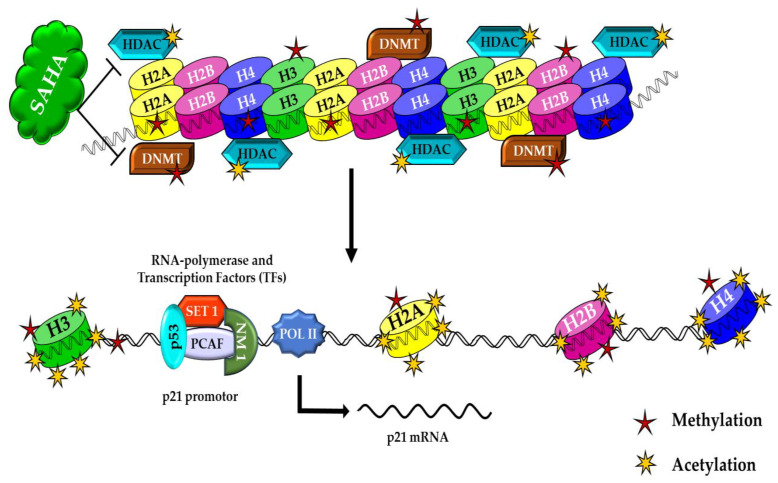
In HDAC inhibitor-treated cancer cells, SAHA helps to augment acetylation, which leads to increased acetylation and decreased methylation of histones. These histone modifications induce higher levels of p21^WAF1/CIP1^ expression in A2780 cancer cells. Upon further stimulation (ex: hyperacetylation), activation of p21^WAF1/CIP1^ gene transcription results in a prolonged retention of cancer cells’ epigenetic alterations.

In the present study, we demonstrated that the HDAC inhibitor SAHA could increase the expression of acetyl histones and decrease DNMTs/HMTs levels in A2780 ovarian cancer cells. In these epigenetics-related modification events, the activity of other proteins, in addition to histones, may also be significantly affected by SAHA. Several studies have shown that the up-regulation and activation of the p53 tumor suppressor protein can lead to cell cycle arrest at the G-to-S transition checkpoint through the induction of p21^WAF1/CIP1^ transcription and also through other mechanisms. Accumulating evidence has implied that the transcriptional stimulation of p21^WAF1/CIP1^ by HDAC inhibitors is activated directly by the hyperacetylation of histones in its promoter region, followed by transcriptional activation through the accelerated binding of transcription factors (TFs) to nucleosomal DNA (Figure 9). In support of this conclusion, recent studies suggest two known mechanisms of epigenetics, i.e., gene inactivation by methylation in the promoter region and the transformation of an active chromatin structure to an inactive form that stops the transcription process. The second mechanism is inhibition of histone deacetylation, which is responsible for the inactivation of *p21^WAF1/CIP1^* transcription, and initiation of gene expression [17]. The p21^WAF1/CIP1^ protein is encoded by the *CDKN1A* gene, and until now, no mutations of the coding region of the *p21^WAF1/CIP1^* gene have been reported in tumor cells [18]. Therefore, hypermethylation of the *p21^WAF1/CIP1^* promoter region may represent an alternative cause by which the *CDKN1A* gene can be inactivated. It has been clearly indicated that the elevation of *p21^WAF1/CIP1^* transcription is primarily through Sp1 and Sp3 promoter sites, in a p53-independent manner in many cells [19]. For example, trichostatin A (TSA) induces the *p21^WAF1/CIP1^* promoter through the Sp1 sites at 282 and 269, relative to the transcription start site in a p53-independent fashion. Sp1 has been shown to repress the transcription of other promoters, such as the murine tyrosine kinase promoter, directly by interaction with HDAC1 [19,20,21,22,23]. It is possible that Sp1 represses *p21^WAF1/CIP1^* transcription also by recruitment of HDACs to the promoter region. However, HDACI may change the balance of histone acetylation status of the *p21^WAF1/CIP1^* promoter at the Sp1 site and increase the expression. Consistent with this speculation, one research group has previously reported that SAHA induces the accumulation of acetylated histones in the chromatin of the *p21^WAF1/CIP1^* gene, which was found to be associated with an increase in transcription [11,24,25].

During our experiments, cell growth inhibition and cell cycle regulation following SAHA treatment were found to be very significant in A2780 ovarian cancer cells. Our results clearly confirmed the down-regulation of both MDMX and MDM2 in A2780 cells, which implies that SAHA treatment can also stop MDM2 and p53 interaction and consequently lead to p21^WAF1/CIP1^ elevation-mediated cell cycle arrest and cell death [8]. However, an acetylation-dependent mechanism is generally considered as responsible for the observed induction of p21^WAF1/CIP1^ expression in cells lacking functional p53. Therefore, the induction of p21^WAF1/CIP1^ during SAHA treatment in many cancer cells is independent of p53, and that may be the dominant mechanism in the A2780 cells used in our experiments. Once elevated, p21^WAF1/CIP1^ is commonly considered responsible for the inhibition of CDK1 and stopping cell division using CDK inhibitory mechanisms [8,26]. In addition, p21^WAF1/CIP1^ is also known to play a significant role in inducing cell death by activating caspases.

SAHA was reported to be more rapid and effective while inducing H2, H3 (K9, K27), and H4 (K8, K12, and K16) hyperacetylation in total cell lysates [27]. A significant elevation of acetyl H2A, H2B, H3, and H4 observed in our experiments, in both the cell lysates as well as in the immunostaining of the whole cells, clearly suggests that induction of p21^WAF1/CIP1^ could be subsequent to the hyperacetylation of histones. While acetyl histone levels were elevated, some methylation-related enzyme levels were markedly down-regulated in A2780 ovarian cancer cells. The *de novo* DNA methylation and adequate methyltransferase levels are crucial for the cells to maintain normal DNA methylation patterns [28,29,30]. Therefore, it can be anticipated that reduced levels of DNMTs/HMTs (DNMT1, DNMT3A, DNMT3B, PRMT1, and SUV39H1) following SAHA treatment can cause hypomethylation of the DNA and histones with or without concomitant demethylation. For example, chronic intermittent ethanol (CIE) exposure was shown to induce upregulation of the N-methyl-D-aspartate (NMDA) receptor and subsequently, a significant decrease in the expression of nine genes coding for enzymes (*EHMT2*, *PRMT6*, *SETDB2*, *SUV39H1*, *SETD1A*, *SETD1B*, *SETD4*, *SETD6*, *and SETDB1*) belonging to the HMTs family in primary cortical neurons [31,32]. Similarly, SAHA appears to reduce the *DNMT3A*, *SUV39H1*, *PRMT7*, *SETD1B*, *SETD7*, *HDAC7*, and *AURKA* levels in A2780 cells, as shown in the gene expression profiler analysis.

In the context of epigenetic therapy, drugs that can modify histone acetylation or DNA methylation are important. Hence, both DNMTIs and HDACIs are in clinical use [17,33,34]; however, the understanding of their mechanistic abilities is constantly growing. When HDACI can open up chromatin by inducing the hyperacetylation of histones, DNMTI, such as 5-aza-deoxycytidine, has been reported to produce genomic DNA hypomethylation [35]. Interestingly some reports have shown that HDACIs and DNMTIs can act synergistically to reactivate genes that are silent and produce effects that can impact the cell cycle and tumor growth [36,37]. TSA has been shown to reactivate methylation-silenced genes by inhibiting HDAC, even in the absence of DNMT inhibitors [38]. In fact, TSA treatment has been reported to decrease DNMTs mRNA levels in endometrial cell lines [28] and DNMT1 enzyme levels in Jurkat T-cells [39,40]. Very similar to our results with A2780 cells, DNMT1 and DNMT3B levels were reported to be decreased when MBA-MD-231 cells were treated with TSA [41,42]. It has also been suggested that HDACI treatment can promote DNMT1 degradation through the ubiquitin-dependent proteasomal pathway, leading to a decrease in DNMT1 levels [43]. A mechanism that was known to induce DNMT1 release was reported to be mediated by p21^WAF1/CIP1^ because it shares the same binding sites with DNMT1 on proliferating cell nuclear antigen (PCNA) [44]. Therefore, it was suspected that p21^WAF1/CIP1^ could bind to PCNA and release DNMT1 for degradation. Thus, TSA treatment was shown to cause genomic hypomethylation as a result of decreased nuclear DNMT1 levels [44] in T24 cells (bladder carcinoma) and also in MDA-MB-231 cells (breast carcinoma) [40].

In general, the changes in the DNA methylation status following HDACI treatment were shown to occur both at the gene-specific locations and also at the global levels to impact multiple genes and their expression status. For example, as discussed above, while down-regulating the methyltransferases levels, the TSA treatment induced decreases in HDAC1, HDAC2, and HDAC3 levels also [17]. Interestingly, in Hep3B cells, HDAC1 was translocated from the nucleus to the cytoplasm, and HDAC2 was also found to be decreased, but they did not find any change in HDAC3 localization following TSA treatment [41]. Apart from the above-discussed changes, in a study conducted with HCT116 cells, PRMT1 levels were also found to be reduced by the propionate, a short-chain fatty acid (SCFA), the treatment that led to the induction of apoptosis following hyperacetylation of histones [45]. Thus, the results from our study conducted with A2780 cells are similar to some of the previous studies that have observed significant decreases in levels of key methyltransferases [42,46,47] when HDACs were inhibited by SAHA or similar drugs. Therefore, it is suspected that these decreases in methyltransferase levels might lead to global DNA/histone hypomethylation, which uniquely coincided with the hyperacetylation of histones in A2780 cells. Hence, the overall epigenetic changes resulting from SAHA treatment might jointly produce cell cycle arrest and cell death through cross-talk at different stages of mechanisms that are related to these cellular events. Altogether, our data suggest that the effects of SAHA are not limited only to hyperacetylation of histones, but might also indirectly impact DNA and histone methylation through down-regulation of epigenetic modifiers such as DNMT1, DNMT3A, DNMT3B, PRMT1, and SUV39H1.

In our studies, SUV39H1 was also significantly down-regulated in A2780 cells after SAHA treatment. SUV39H1 is an important methyltransferase in humans, responsible for dimethylation and trimethylation (H3K9me2/3) of H3 at lysine 9 [48,49,50]. Previous studies have shown that overexpression of SUV39H1 drove tumorigenesis in mice, resulting in a significant decrease in survival rates [51]. Thus, there is sufficient evidence to show that SUV39H1 is involved in regulating tumor growth, metastasis, and cell proliferation. Interestingly, SAHA treatment was able to significantly reduce the expression of SUV39H1 in A2780 cells during our experiments. The mechanisms underlying the down-regulation of SUV39H1 may not be a cell-specific effect, because similar down-regulation of mRNA levels of *SUV39H1* also was observed in other cancer cells such as HCC827, H460, LNCaP, and MCF-7 (data not shown). Another important class of proteins altered by SAHA treatment are the Aurora kinases (AURKs), which play a vital role in the cell-cycle control pathway. The overexpression or gene amplification of AURKs have been explained in various malignant growths. In many tissues, Aurora kinase overexpression leads to genetic instability (aneuploidy), which is known to cause cancer. Recently, it has been shown that AURKA is overexpressed and involved in tumorigenesis through multiple mechanisms [52]. AURKA can phosphorylate RAS association domain family 1 isoform A (RASSF1A) protein, which is a novel tumor suppressor. However, phosphorylation of RASSF1A is shown to disturb microtubule equilibrium and cell cycle regulation, which can eventually lead to uncontrolled proliferation in cancers [53]. AURKB has been shown to diminish the expression of p21^WAF1/CIP1^ through inhibition of p53 activity [54], resulting in aberrant activation of CDK1, which can eventually lead to cell cycle progression and survival of cancer cells. Furthermore, overexpression of AURKC has also been detected in human colorectal cancers, thyroid carcinoma, and several cancer cells. Although, the regulation and clinical implications of overexpressed AURKC in cancer cells are unclear, elevated levels of this enzyme were suspected to increase the proliferation, transformation, and migration of cancer cells [55]. In the present study, HDAC inhibition by SAHA was able to significantly down-regulate the mRNA levels of *AURKA*, *AURKB*, and *AURKC* in A2780 cells. We have previously noted an intracellular link between AURKB and MDM2 in LNCaP cells [56]. We have also shown that, in A2780 cells, inhibition of MDM2 with RG7388 was also able to reduce the levels of AURKB and CDC25C significantly. Consequently, it was suspected that p21^WAF1/CIP1^ and p27^Kip1^ might mediate the decrease in AURK levels [8]. So far, it is not clear whether the effects of SAHA on the AURK family of proteins are due to a direct effect on their transcriptional machinery or through some other mechanisms involving p21^WAF1/CIP1^. However, it is clear that the impact of HDAC inhibition on AURKA, AURKB, and AURKC expression is significant and should be used as an additive strategy for inducing cell cycle arrest and cell death in cancers where the AURK family of proteins are elevated.

Several of the above-discussed results were found in A2780 cancer cells that were growing not only as monolayers, but also as spheroids. The cells growing as spheroids are morphologically similar to tumors that grow inside the body and mimic the tumor microenvironment (TME). The SAHA-induced histone acetylations (H2A, H2B, H3, and H4) in both the spheroid model as well as the monolayer of A2780 cells allow prediction of the possibility of similar occurrences in tumors growing inside the body.

## 5. Conclusions

In conclusion, our results demonstrated significant down-regulation of methyltransferase enzyme levels in A2780 ovarian cancer cells, which is suspected to impact global methylation status and consequently gene expression, while histones exhibited hyperacetylation following the inhibition of HDACs with SAHA.

## Figures and Tables

**Table 1:** List of selected up- and down-regulated genes modulated by suberoylanilide hydroxamic acid (SAHA) treatment in A2780 ovarian cancer cells analyzed using PCR array.

Gene	Accession Number	Description	Fold Change
**Up-regulated genes**			
*RPS6KA5*	NM_004755	Ribosomal protein S6 kinase, 90 kDa, polypeptide 5	1.96
*HDAC3*	NM_003883	Histone deacetylase 3	1.79
*HDAC9*	NM_178425	Histone deacetylase 9	1.7
*SETD4*	NM_017438	Su(var)3–9, enhancer-of-zeste, trithorax (SET) domain containing 4	1.78
*SMYD3*	NM_022743	SET and Myeloid-Nervy-DEAF1 (MYND) domain containing 3	1.6
*HDAC4*	NM_006037	Histone deacetylase 4	1.23
**Down-regulated genes**			
*SETD1B*	NM_015048	SET domain containing 1B	0.24
*HDAC7*	NM_001098416	Histone deacetylase 7	0.27
*NSD1*	NM_022455	Nuclear receptor binding SET, domain protein 1	0.29
*DNMT3A*	NM_022552	DNA (cytosine-5-)-methyltransferase 3 alpha	0.31
*WHSC1*	NM_007331	Wolf-Hirschhorn syndrome candidate 1	0.35
*AURKA*	NM_003600	Aurora kinase A	0.41
*EHMT2*	NM_006709	Euchromatic histone-lysine N-methyltransferase 2	0.41
*KAT7*	NM_007067	K(lysine) acetyltransferase 7	0.43
*SUV39H1*	NM_003173	Suppressor of variegation 3–9 homolog 1 (Drosophila)	0.43
*NACOA6*	NM_014071	Nuclear receptor coactivator 6	0.44
*SETD7*	NM_030648	SET domain containing (lysine methyltransferase) 7	0.44
*PRMT7*	NM_019023	Protein arginine methyltransferase 7	0.49
*CARM1*	NM_199141	Coactivator-associated arginine methyltransferase 1	0.51
*KAT8*	NM_032188	K(lysine) acetyltransferase 8	0.53
*KMT2E*	NM_182931	Myeloid/lymphoid or mixed-lineage leukemia 5 (trithorax homolog, Drosophila)	0.54
*NEK6*	NM_014397	NIMA (never in mitosis gene a) -related kinase 6	0.54
*KMT2A*	NM_005933	Myeloid/lymphoid or mixed-lineage leukemia (trithorax homolog, Drosophila)	0.56
*KAT6B*	NM_012330	K(lysine) acetyltransferase 6B	0.57
*SETD5*	NM_001080517	SET domain containing 5	0.57
*AURKC*	NM_003160	Aurora kinase C	0.58
*AURKB*	NM_004217	Aurora kinase B	0.59
*PRMT1*	NM_001536	Protein arginine methyltransferase 7	0.61
*HDAC2*	NM_001527	Histone deacetylase 2	0.75

## Data Availability

Data available under request to the corresponding author of the study.
